# Development and validation of a nomogram for differentiating granulomatous lobular mastitis from ductal carcinoma in situ

**DOI:** 10.3389/fonc.2025.1668908

**Published:** 2025-12-08

**Authors:** Youjia Li, Liyang Su, Qingquan Zhang, Zhonghua Liu

**Affiliations:** Department of Ultrasound, Quanzhou First Hospital, Quanzhou, China

**Keywords:** granulomatous mastitis, carcinoma, intraductal, noninfiltrating, nomograms, ultrasonography, mammary, predictive learning models

## Abstract

**Background:**

Granulomatous lobular mastitis (GLM) frequently mimics ductal carcinoma *in situ* (DCIS) in clinical presentation and imaging characteristics, leading to misdiagnosis and unnecessary aggressive interventions. This study aimed to develop and validate a practical nomogram for differentiating GLM from DCIS.

**Methods:**

We conducted a retrospective study at Quanzhou First Hospital from January 2020 to April 2025, including 290 patients with histopathologically confirmed GLM (n=128) or DCIS (n=162). Patients were randomly divided into training (n=203) and validation (n=87) sets. Clinical, laboratory, and ultrasound features were analyzed using univariate and multivariate logistic regression to identify independent predictors. A nomogram was constructed and evaluated using receiver operating characteristic (ROC) curves, calibration plots, and decision curve analysis.

**Results:**

Six independent predictors were incorporated into the final nomogram: age, lesion size, margin characteristics, microcalcifications, posterior acoustic enhancement, and peri-lesional flow. The nomogram demonstrated excellent discriminative performance with areas under the ROC curve of 0.95 (95% CI: 0.92-0.98) in the training set and 0.93 (95% CI: 0.88-0.98) in the validation set. At optimal thresholds, the model achieved sensitivity of 92% and specificity of 89% in the training set, and 89% and 79% respectively in the validation set. Calibration plots confirmed high predictive accuracy, and decision curve analysis demonstrated substantial clinical benefit across clinically relevant threshold probabilities.

**Conclusions:**

This novel nomogram represents a diagnostic tool specifically designed for GLM versus DCIS differentiation. Its reliance on widely available clinical and ultrasound parameters makes it particularly valuable for resource-limited settings, potentially reducing unnecessary biopsies and associated patient morbidity.

## Introduction

Granulomatous lobular mastitis (GLM) is a rare, chronic inflammatory breast condition primarily affecting women of reproductive age, with a global prevalence of approximately 2.4 per 100,000 women (1, 2). Characterized by non-caseous granulomatous inflammation of the breast lobules, GLM often mimics breast malignancy, presenting with palpable masses, skin changes, and imaging abnormalities (3, 4). This mimicry leads to frequent misdiagnosis as ductal carcinoma *in situ* (DCIS) or invasive breast cancer, resulting in unnecessary mastectomies, chemotherapy, or radiation therapy, which cause significant patient morbidity and healthcare costs (5). Accurate differentiation of GLM from DCIS, which accounts for 25% of breast cancer diagnoses (over 56,000 annual cases in the United States) (6), is critical yet challenging due to overlapping clinical and imaging features, including irregular masses and architectural distortion (7).

Current diagnostic approaches, including the Breast Imaging-Reporting and Data System (BI-RADS) ultrasound classification, mammography, and MRI, rely on histopathological confirmation via core needle biopsy, as imaging alone lacks specificity for GLM (4). BI-RADS often misclassifies GLM as category 4 or 5 lesions, triggering invasive procedures. Recent studies have developed MRI-based radiomics models for differentiating granulomatous mastitis from breast malignancies, utilizing texture analysis and machine learning algorithms with promising results (8). However, these radiomics approaches, along with other MRI and deep learning-based radiomics models, though promising, are costly and inaccessible in resource-limited settings (9, 10). Existing nomograms focus on malignancy detection or differentiation between invasive and non-invasive cancers, neglecting GLM’s unique inflammatory characteristics (11). This diagnostic gap underscores the need for a tailored, accessible tool to improve GLM identification and reduce misdiagnosis.

Nomograms, as predictive models integrating multiple clinical parameters, offer a practical solution for complex differential diagnoses (12). This study addresses the diagnostic challenge by developing and validating a practical nomogram specifically designed to differentiate GLM from DCIS using readily available clinical and ultrasound parameters. By integrating clinical, ultrasound, and laboratory parameters, our model aims to enhance diagnostic accuracy, reduce unnecessary invasive procedures, and support conservative management in resource-constrained settings, ultimately improving patient outcomes.

## Materials and methods

### Study design and ethical considerations

This retrospective cohort study was conducted at Quanzhou First Hospital Affiliated to Fujian Medical University from January 2020 to April 2025. The study protocol was approved by the Institutional Review Board of Quanzhou First Hospital (Ethics Approval No: Quanyi Lun [2025]K152), and informed consent was waived due to the retrospective design, in accordance with the Declaration of Helsinki.

### Patient selection

**Figure 1:**
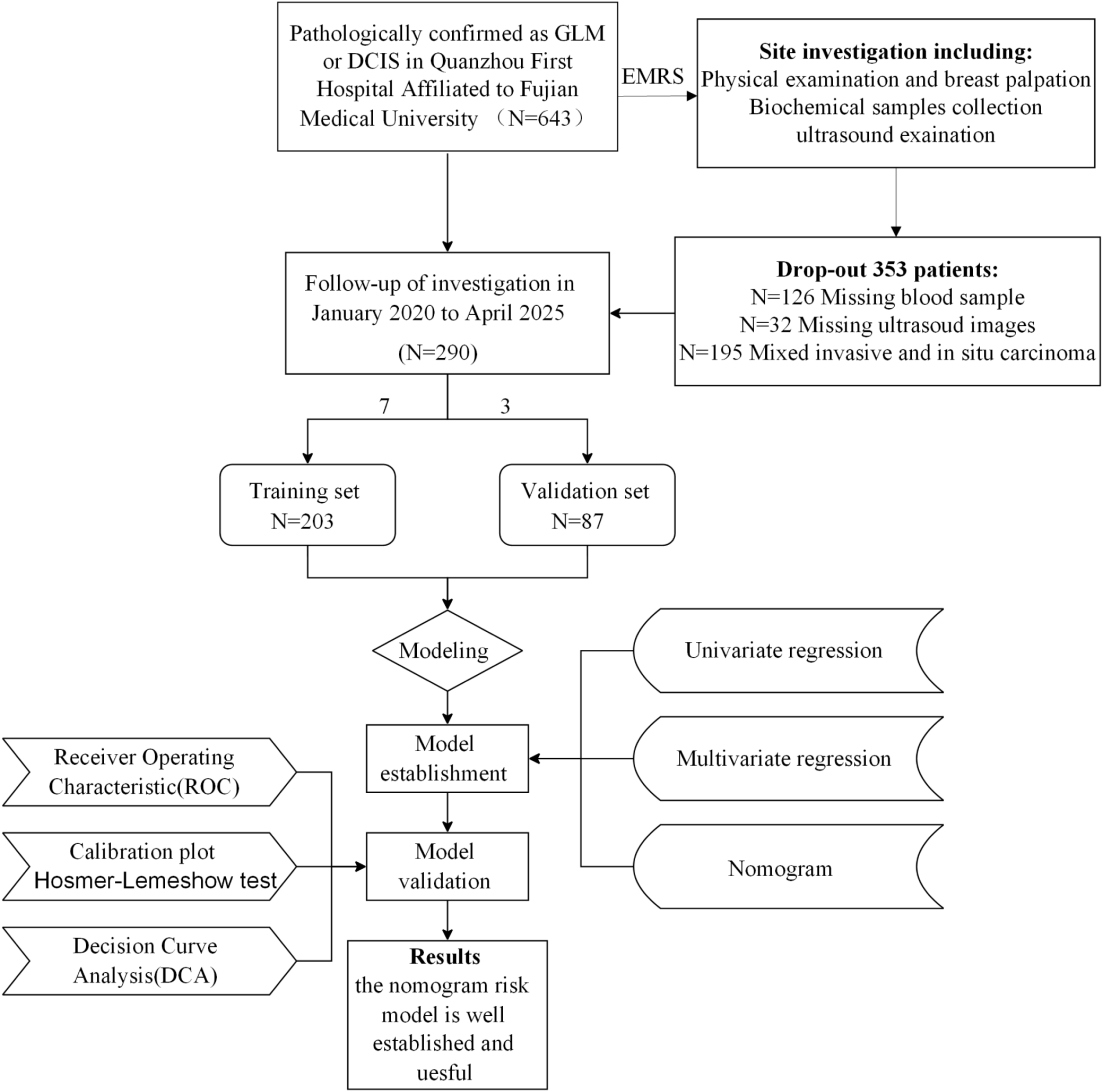
Patient selection flowchart and study design. A total of 643 patients with pathologically confirmed GLM or DCIS were initially enrolled from Quanzhou First Hospital Affiliated to Fujian Medical University. After excluding 353 patients due to various criteria (missing or inadequate ultrasound images, missing samples, and presence of mixed invasive and *in situ* carcinoma), 290 patients were included in the final analysis and randomly divided into training (n=203) and validation (n=87) sets. The study workflow included data collection, model establishment using univariate and multivariate regression analysis, and model validation through ROC analysis, calibration plots, Hosmer-Lemeshow test, and decision curve analysis.

Patients with pathologically confirmed granulomatous lobular mastitis (GLM) or ductal carcinoma *in situ* (DCIS) were identified via the hospital’s electronic medical record system (EMRS). GLM was diagnosed based on World Health Organization (WHO) criteria, requiring non-caseous granulomatous inflammation of breast lobules (13). DCIS was confirmed per WHO breast tumor classification, characterized by absence of stromal invasion (14). Inclusion criteria were: (1) histopathologically confirmed GLM or DCIS via core needle biopsy or surgical specimen; (2) complete clinical, laboratory, and ultrasound data; (3) high-quality breast ultrasound images; (4) age ≥18 years; (5) complete follow-up data. Exclusion criteria included: (1) missing or inadequate ultrasound images (n=32); (2) missing blood sample data (n=126); (3) presence of mixed invasive and *in situ* carcinoma (n=195). Of 643 initial patients, 290 were included (162 DCIS, 128 GLM) and randomly allocated to training (n=203; 118 DCIS, 85 GLM) and validation (n=87; 44 DCIS, 43 GLM) sets using R software (version 4.3.0, sample function) with a random seed of 1256 to minimize selection bias (**Figure 1**).

### Data collection

Clinical data, including age, height, weight, white blood cell count (WBC), carcinoembryonic antigen (CEA), CA125, CA153, and palpation findings, were extracted from EMRS by two trained researchers, with double-entry verification to ensure accuracy. These tumor markers were included based on their potential relevance in differentiating inflammatory from neoplastic breast conditions: CEA as a broad-spectrum tumor marker potentially elevated in malignant conditions, CA125 which may be elevated in inflammatory conditions like GLM due to inflammatory response, and CA153 as a breast-specific tumor marker theoretically more relevant to DCIS. Laboratory parameters were collected within 48 hours of ultrasound examination. Breast ultrasound was performed using Philips EPIQ Q5/Q7 or Siemens ACUSON Sequoia systems with 5–12 MHz linear transducers, calibrated per manufacturer guidelines. Patients were positioned supine with arms elevated, and both breasts were scanned in sagittal and transverse planes. Images were stored in DICOM format and reviewed by a senior radiologist to confirm adequate visualization of lesion characteristics.

### Ultrasound evaluation

Ultrasound images were independently assessed by two breast radiologists (8 and 12 years of experience) blinded to clinical and histopathological data, with discrepancies resolved by consensus or a third senior radiologist. Lesion characteristics were evaluated per the BI-RADS ultrasound lexicon, including shape, aspect ratio, margins (distinct vs. indistinct), architectural distortion, microcalcifications, posterior acoustic features (enhancement vs. other), internal and peri-lesional flow, lymph node status, and focus number (single vs. multiple), as detailed in **Table 1**.

**Table 1:** Baseline characteristics in the training and validation sets.

Variables	Training set			Validation set		
	DCIS (n = 118)	GLM (n = 85)	P	DCIS (n = 44)	GLM (n = 43)	P
Age (years)	47.14 ± 9.00	36.12 ± 7.56	<.001	49.39 ± 9.23	36.79 ± 7.34	<.001
Height (cm)	157.85 ± 5.01	158.77 ± 4.94	0.254	157.07 ± 4.80	158.28 ± 5.66	0.308
Weight (kg)	62.68 ± 46.51	62.72 ± 11.23	0.995	56.26 ± 8.57	63.38 ± 10.04	0.001
WBC (10^9/L)	7.07 ± 1.81	9.85 ± 6.69	<.001	7.48 ± 2.37	8.74 ± 3.08	0.035
CEA (mg/dl)	1.90 ± 1.46	1.81 ± 2.43	0.771	2.12 ± 1.46	1.53 ± 1.07	0.058
CA125 (U/mL)	18.84 ± 22.11	19.52 ± 10.74	0.855	14.46 ± 8.85	23.67 ± 18.60	0.016
CA153 (U/mL)	12.55 ± 18.64	9.52 ± 5.41	0.326	13.92 ± 17.66	9.23 ± 4.87	0.162
Size (cm)	2.41 ± 1.73	3.31 ± 1.41	<.001	2.55 ± 1.71	3.31 ± 1.69	0.040
Palpation, n(%)			0.656			0.756
Palpable	26 (22.03)	21 (24.71)		10 (22.73)	11 (25.58)	
Unpalpable	92 (77.97)	64 (75.29)		34 (77.27)	32 (74.42)	
Shape, n(%)			0.087			0.313
Regular	19 (16.10)	22 (25.88)		4 (9.09)	7 (16.28)	
Irregular	99 (83.90)	63 (74.12)		40 (90.91)	36 (83.72)	
Aspect ratio, n(%)			0.400			0.592
Aspect ratio <1	115 (97.46)	80 (94.12)		43 (97.73)	40 (93.02)	
Aspect ratio ≥1	3 (2.54)	5 (5.88)		1 (2.27)	3 (6.98)	
Margin, n(%)			<.001			<.001
Distinct	29 (24.58)	43 (50.59)		6 (13.64)	20 (46.51)	
Indistinct	89 (75.42)	42 (49.41)		38 (86.36)	23 (53.49)	
Architectural dist, n(%)			0.045			1.000
No	117 (99.15)	79 (92.94)		41 (93.18)	40 (93.02)	
Yes	1 (0.85)	6 (7.06)		3 (6.82)	3 (6.98)	
Microcalcification, n(%)			<.001			<.001
Absent	47 (39.83)	79 (92.94)		19 (43.18)	38 (88.37)	
Present	71 (60.17)	6 (7.06)		25 (56.82)	5 (11.63)	
Posterior echo, n(%)			<.001			<.001
Other echoes	96 (81.36)	29 (34.12)		41 (93.18)	20 (46.51)	
Enhancement	22 (18.64)	56 (65.88)		3 (6.82)	23 (53.49)	
Internal flow, n(%)			0.131			0.598
Absent	57 (48.31)	32 (37.65)		18 (40.91)	20 (46.51)	
Present	61 (51.69)	53 (62.35)		26 (59.09)	23 (53.49)	
Peri flow, n(%)			0.011			0.299
Absent	57 (48.31)	26 (30.59)		18 (40.91)	13 (30.23)	
Present	61 (51.69)	59 (69.41)		26 (59.09)	30 (69.77)	
Lymph node, n(%)			0.172			0.230
Normal	83 (70.34)	52 (61.18)		31 (70.45)	25 (58.14)	
Abnormal	35 (29.66)	33 (38.82)		13 (29.55)	18 (41.86)	
Focus num, n(%)			0.111			0.031
Single	86 (72.88)	53 (62.35)		35 (79.55)	25 (58.14)	
Multiple	32 (27.12)	32 (37.65)		9 (20.45)	18 (41.86)	

### Statistical analysis

**Figure 2:**
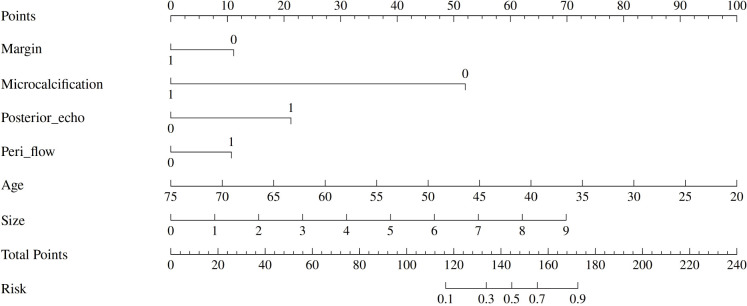
Nomogram for differentiating GLM from DCIS. The nomogram incorporates six clinical and imaging variables: margin characteristics, microcalcification presence, posterior acoustic enhancement, peri-lesional flow, patient age, and lesion size. Each variable contributes points based on its value, with the total points corresponding to the predicted risk of DCIS. The nomogram provides a practical tool for clinical decision-making in differential diagnosis.

**Figure 3:**
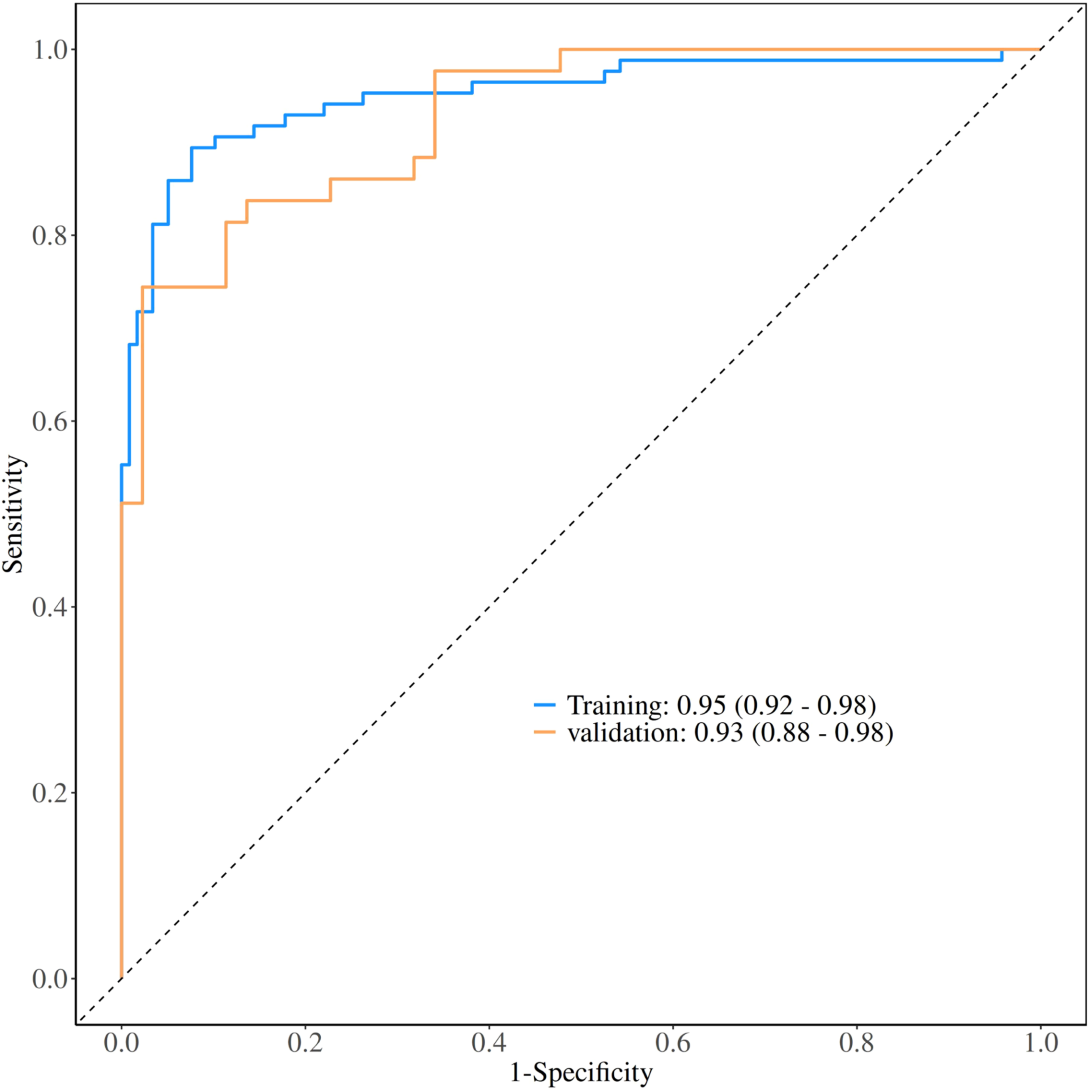
Receiver operating characteristic (ROC) curves for model performance evaluation. The ROC curves demonstrate the diagnostic performance of the nomogram model in both training and validation datasets. The area under the curve (AUC) values were 0.95 (95% CI: 0.92-0.98) for the training set and 0.93 (95% CI: 0.88-0.98) for the validation set, indicating excellent discriminative ability.

**Figure 4:**
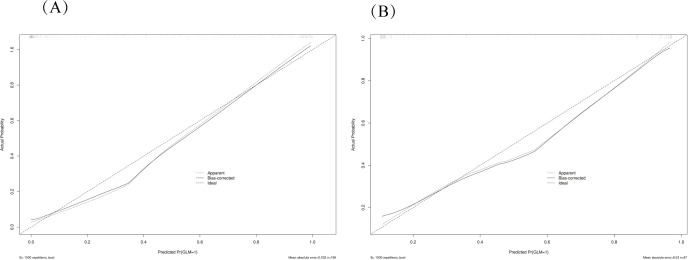
Calibration plots for model validation. The calibration plots show the agreement between predicted probabilities and observed outcomes in both training **(A)** and validation **(B)** datasets. The diagonal line represents perfect calibration, while the plotted line shows the model’s actual performance. The close alignment indicates good calibration of the nomogram model.

**Figure 5:**
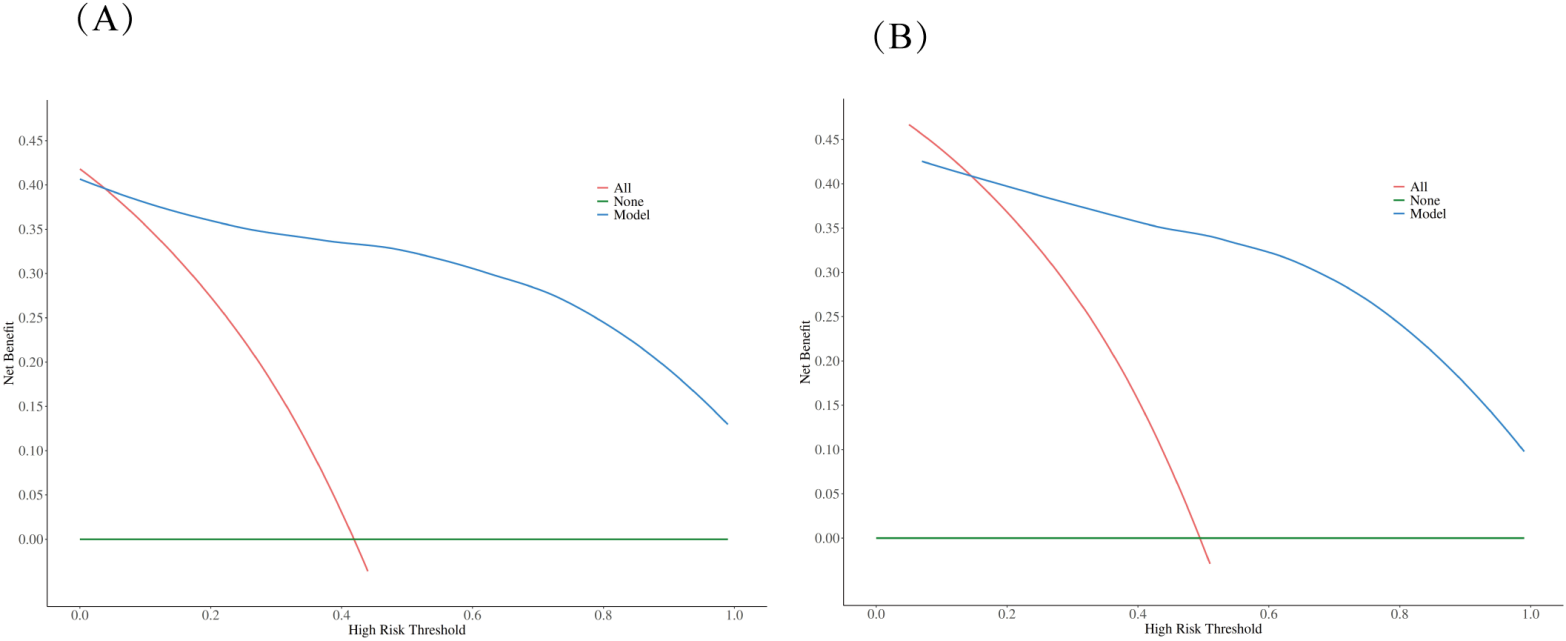
Decision curve analysis (DCA) comparing clinical utility. The DCA evaluates the clinical usefulness of the nomogram model compared to treating all patients or no patients across different risk thresholds in both training **(A)** and validation **(B)** datasets. The analysis demonstrates that the nomogram model provides superior net benefit over the “treat all” or “treat none” strategies across a wide range of threshold probabilities, supporting its clinical utility in decision-making.

Continuous variables (e.g., age, WBC, lesion size) were reported as mean ± standard deviation, and categorical variables (e.g., margins, microcalcifications) as frequencies (percentages). Receiver operating characteristic (ROC) curve analysis was performed to determine the optimal cut-off value for WBC in differentiating GLM from DCIS, with the Youden index used to identify the optimal threshold. Baseline characteristics were compared using t-tests or Mann-Whitney U tests for continuous variables and chi-square tests for categorical variables (**Table 1**). Missing blood sample data (n=126) were excluded after sensitivity analysis confirmed no significant bias. Univariate logistic regression identified eight significant predictors (p<0.05): age, white blood cell count (WBC), lesion size, margin characteristics, architectural distortion, microcalcifications, posterior acoustic enhancement, and peri-lesional flow. ROC curve analysis for WBC revealed an optimal cut-off value of 7.735 × 10^9^/L with sensitivity of 68% (95% CI: 61%-76%), specificity of 65% (95% CI: 57%-74%), and AUC of 0.69 (95% CI: 0.63-0.75) for differentiating GLM from DCIS. Multivariate logistic regression with backward stepwise selection (entry p<0.05, removal p>0.10) was performed to construct the nomogram, starting with an AIC of 127.74 and reducing to 127.29 after excluding WBC and architectural distortion. Although WBC showed moderate discriminatory ability (AUC = 0.69) with statistical significance in univariate analysis, it was excluded from the final multivariable model due to its relatively weaker predictive contribution compared to imaging features, and to maintain model parsimony while optimizing overall performance (VIF: WBC 1.183, architectural distortion 1.066). The final model incorporated six independent predictors: age, lesion size, margins, microcalcifications, posterior acoustic enhancement, and peri-lesional flow (**Figure 2**). Multicollinearity was assessed using variance inflation factors (VIF), with values for the final model ranging from 1.176 to 1.399, indicating no significant collinearity. Model performance was evaluated using receiver operating characteristic (ROC) curves (area under the curve [AUC] 0.95 [95% CI: 0.92–0.98] for training, 0.93 [95% CI: 0.88–0.98] for validation; **Figure 3**), calibration plots with the Hosmer-Lemeshow test (**Figure 4**), and decision curve analysis (DCA) across threshold probabilities of 0.1–0.9 (**Figure 5**). Internal validation was conducted using 1000 bootstrap resamples to assess overfitting. All analyses were performed in R (version 4.3.0) using packages rms, pROC, and dca. Statistical significance was set at p<0.05.

## Results

### Patient characteristics

A total of 290 patients were included in this retrospective cohort study, comprising 203 patients in the training set and 87 patients in the validation set. Patients with DCIS were significantly older than those with GLM in both training and validation sets (P < 0.001), while height showed no significant differences between groups. White blood cell counts were significantly elevated in GLM patients compared to DCIS patients in both cohorts (P < 0.05). ROC curve analysis for WBC demonstrated an area under the curve (AUC) of 0.69 (95% CI: 0.63-0.75, P<0.001). The optimal cut-off value was determined to be 7.735 × 10^9^/L, yielding a sensitivity of 68% (95% CI: 61%-76%), specificity of 65% (95% CI: 57%-74%), accuracy of 67% (95% CI: 61%-72%), positive predictive value of 72% (95% CI: 65%-79%), and negative predictive value of 61% (95% CI: 53%-70%) for GLM diagnosis. Lesion size was significantly larger in GLM patients in both training and validation sets (P < 0.05). Several ultrasound characteristics showed significant inter-group differences: GLM lesions more frequently exhibited distinct margins compared to DCIS lesions (P<0.001), while microcalcifications were markedly more frequent in DCIS patients (P < 0.001). Posterior acoustic enhancement was significantly more common in GLM lesions (P < 0.001), and peripheral flow was more frequent in GLM patients in the training set P < 0.05). These baseline characteristics demonstrate distinct clinical and ultrasound profiles between DCIS and GLM, providing the foundation for the development of predictive models in this study. Baseline characteristics were well-balanced between training and validation sets, confirming study reliability, with detailed patient characteristics presented in **Table 1**.

### Logistic regression analysis in the training set and construction of nomogram

To identify independent predictors for differentiating DCIS from GLM, univariate and multivariate logistic regression analyses were performed using the training set data. The detailed results of univariate and multivariate analyses are presented in **Table 2**. In the univariate analysis, several variables demonstrated significant associations with the diagnosis: margin characteristics (P < 0.001), architectural distortion (P = 0.045), microcalcification (P < 0.001), posterior echo enhancement (P < 0.001), peripheral flow (P = 0.012), age (P < 0.001), white blood cell count (P < 0.001), and lesion size (P < 0.001).

**Table 2:** Univariate and multivariate logistic regression analysis in the training set.

Characteristics	Univariate analysis		Multivariate analysis	
	*P*	OR (95%CI)	*P*	OR (95%CI)
Age	<.001	0.85 (0.81 ~ 0.89)	<.001	0.87 (0.82 ~ 0.92)
Height	0.254	1.04 (0.97 ~ 1.11)	NA	NA
Weight	0.995	1.00 (0.99 ~ 1.01)	NA	NA
WBC	<.001	1.48 (1.27 ~ 1.73)	NA	NA
CEA	0.77	0.97 (0.77 ~ 1.21)	NA	NA
CA125	0.854	1.00 (0.98 ~ 1.02)	NA	NA
CA153	0.365	0.98 (0.92 ~ 1.03)	NA	NA
Size	<.001	1.41 (1.17 ~ 1.69)	0.001	1.84 (1.26 ~ 2.67)
Palpation	0.656	0.86 (0.45 ~ 1.66)	NA	NA
Shape	0.089	0.55 (0.28 ~ 1.10)	NA	NA
Aspect ratio	0.241	2.40 (0.56 ~ 10.31)	NA	NA
Margin	<.001	0.32 (0.18 ~ 0.58)	0.092	0.42 (0.15 ~ 1.15)
Architectural dist	0.045	8.89 (1.05 ~ 75.24)	NA	NA
Microcalcification	<.001	0.05 (0.02 ~ 0.12)	<.001	0.02 (0.00 ~ 0.08)
Posterior echo	<.001	8.43 (4.42 ~ 16.06)	0.002	5.28 (1.83 ~ 15.29)
Internal flow	0.132	1.55 (0.88 ~ 2.73)	NA	NA
Peri flow	0.012	2.12 (1.18 ~ 3.81)	0.096	2.31 (0.86 ~ 6.20)
Lymph node	0.173	1.50 (0.84 ~ 2.71)	NA	NA
Focus num	0.112	1.62 (0.89 ~ 2.95)	NA	NA

NA, Not Applicable.

Subsequently, multivariate logistic regression analysis was performed using backward stepwise selection to identify independent predictors. All eight variables that demonstrated statistical significance in univariate analysis (p < 0.05) were initially included in the full multivariable logistic regression model. Using a removal criterion of p > 0.10, non-significant variables were sequentially eliminated: architectural distortion was first removed (p = 0.382), followed by white blood cell count (p = 0.199). Multicollinearity was assessed using variance inflation factors (VIF), confirming no multicollinearity issues (all VIF < 1.4). The final model achieved an AIC value of 127.29, representing an improvement over the full model (AIC = 127.74), indicating good model fit and clinical utility.

The final multivariate model identified six independent predictors that were retained in the nomogram: margin characteristics, microcalcification, posterior echo enhancement, peripheral flow, age, and lesion size. Based on these independent predictors, a predictive nomogram was constructed (**Figure 2**) to provide a visual and practical tool for clinical decision-making. The nomogram incorporates a point-based scoring system where each variable contributes specific points based on its value. The total points range from 0 to 240, corresponding to GLM risk probabilities from 0.1 to 0.9. To use the nomogram, clinicians locate each variable’s value on its respective axis, draw a vertical line to the points axis to determine the corresponding points, sum all points to obtain the total score, and finally project this total score onto the risk axis to estimate the probability of GLM diagnosis. This user-friendly tool enables individualized risk assessment and facilitates clinical decision-making in distinguishing between DCIS and GLM based on readily available clinical and ultrasound parameters.

### Evaluation of nomogram

The performance of the constructed nomogram was comprehensively evaluated using multiple statistical metrics in both training and validation sets. Discrimination ability was assessed using receiver operating characteristic (ROC) curve analysis, which demonstrated excellent predictive performance with an area under the curve (AUC) of 0.95 (95% CI: 0.92-0.98) in the training set and 0.93 (95% CI: 0.88-0.98) in the validation set (**Figure 3**). These high AUC values indicate superior discriminatory capacity of the nomogram in distinguishing between DCIS and GLM. Calibration performance was evaluated using calibration plots, which showed good agreement between predicted probabilities and observed outcomes in both training and validation sets (**Figure 4**). The calibration curves demonstrated that the nomogram-predicted probabilities closely aligned with the actual probabilities, with minimal deviation from the ideal reference line and mean absolute errors of 0.032 and 0.03 for the training and validation sets, respectively. Bias-corrected calibration curves further confirmed the model’s reliability and suggested minimal overfitting. Clinical utility was assessed through decision curve analysis (DCA), which evaluates the net benefit of using the nomogram across different threshold probabilities. The DCA demonstrated that the nomogram provided substantial clinical benefit compared to treating all patients or treating no patients across a wide range of threshold probabilities (**Figure 5**). In both training and validation sets, the nomogram consistently outperformed the “treat all” and “treat none” strategies, with net benefit curves remaining above the reference lines across most clinically relevant threshold probabilities. These results collectively indicate that the developed nomogram exhibits excellent discrimination, good calibration, and meaningful clinical utility, making it a reliable tool for differentiating DCIS from GLM in clinical practice.

**Figure 6:**
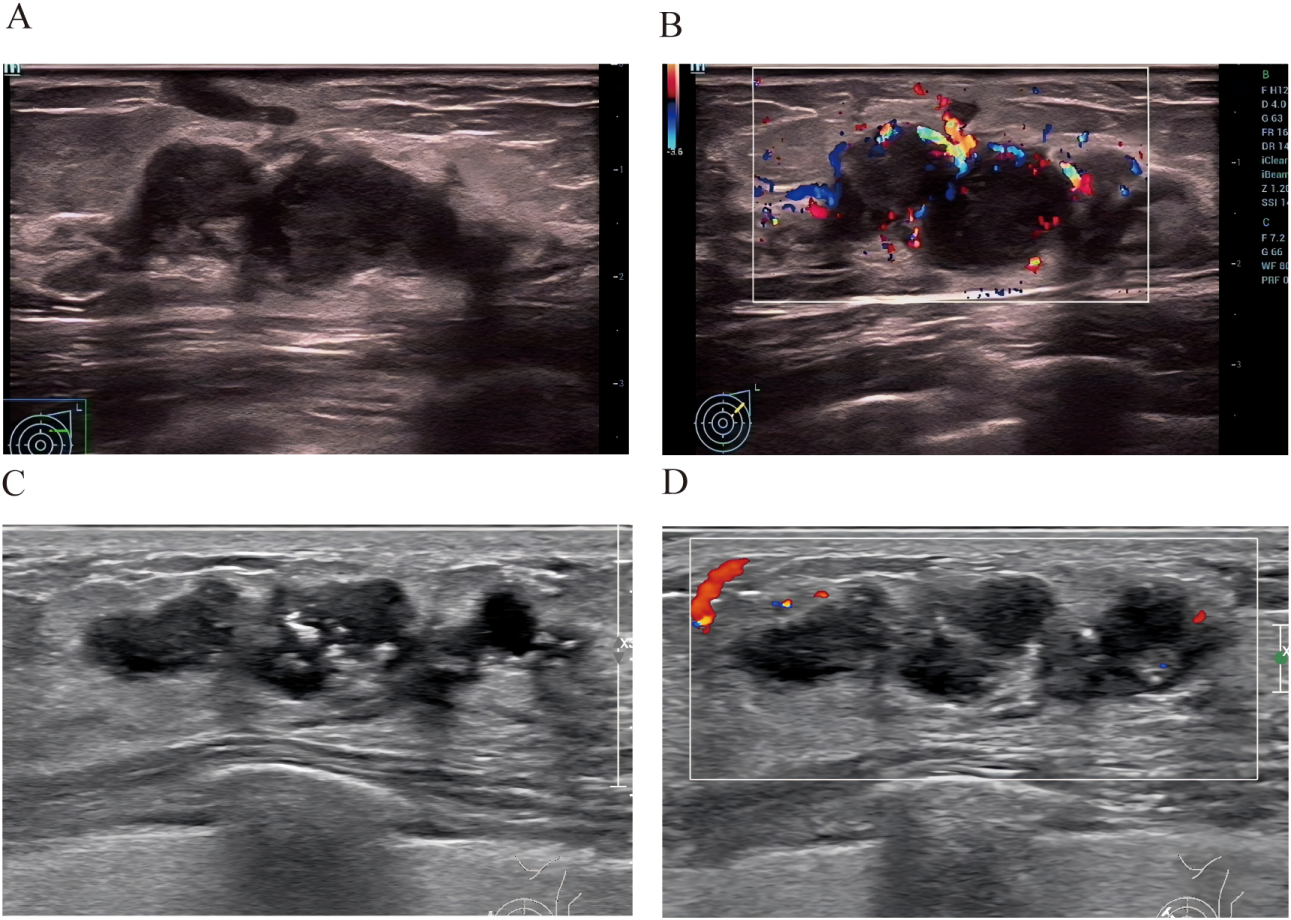
Two cases whose outcomes were successfully predicted by the nomogram model. Case 1 **(A, B)** A 38-year-old female patient presented with ultrasound characteristics of indistinct margins, absence of microcalcifications, posterior acoustic enhancement, peri-lesional flow, and a lesion size of 3.2 cm, with a predicted probability of 90%.Case 2 **(C, D)** A 45-year-old female patient exhibited ultrasound findings of indistinct margins, presence of microcalcifications, absence of posterior acoustic enhancement, peri-lesional flow, and a lesion size of 3.4 cm, with a predicted probability of 10%.

Two cases whose outcomes were successfully predicted by the nomogram model(**Figure 6**). Case 1 (**Figures 6A, B**) A 38-year-old female patient presented with ultrasound characteristics of indistinct margins, absence of microcalcifications, posterior acoustic enhancement, peri-lesional flow, and a lesion size of 3.2 cm, with a predicted probability of 90%. Case 2 (**Figures 6C, D**) A 45-year-old female patient exhibited ultrasound findings of indistinct margins, presence of microcalcifications, absence of posterior acoustic enhancement, peri-lesional flow, and a lesion size of 3.4 cm, with a predicted probability of 10%.

## Discussion

Granulomatous lobular mastitis (GLM) remains a crucial and underrecognized diagnostic challenge in breast pathology, often imitating breast carcinoma due to its inflammatory presentation and overlapping imaging features (4, 15). As highlighted in recent literature, GLM’s mimicry of malignancy, including ductal carcinoma *in situ* (DCIS), underscores its clinical importance, leading to frequent misdiagnoses and unnecessary aggressive interventions such as mastectomies or chemotherapy (5, 16).This issue is particularly pertinent in reproductive-age women, where GLM’s idiopathic nature and potential links to immune dysregulation or hormonal factors amplify diagnostic complexity (17, 18). The developed nomogram, integrating age, lesion size, margin characteristics, microcalcifications, posterior acoustic enhancement, and peri-lesional flow, represents a pioneering tool for differentiating granulomatous lobular mastitis (GLM) from ductal carcinoma *in situ* (DCIS), achieving an area under the curve (AUC) of 0.95 in the training set and 0.93 in the validation set. This model’s clinical utility is underscored by its high sensitivity (0.92/0.89) and specificity (0.89/0.79), surpassing the typical specificity of BI-RADS ultrasound (0.60–0.70) (19, 20). This improvement may translate to a significant reduction in unnecessary biopsies, as suggested by the validation set’s enhanced specificity (0.79 vs. 0.60–0.70 for BI-RADS), providing substantial benefits in decreasing patient morbidity and healthcare costs associated with misdiagnosis-driven interventions, such as mastectomies or chemotherapy (5).

The selected predictors provide distinct diagnostic insights. Age and lesion size reflect the contrasting pathological mechanisms, with older age favoring DCIS due to its neoplastic nature (6, 21), while larger lesion sizes are more indicative of GLM’s inflammatory progression (3, 22). Ultrasound features like indistinct margins and microcalcifications are strongly associated with DCIS’s malignant characteristics (23, 24), whereas posterior acoustic enhancement and peri-lesional flow highlight GLM’s inflammatory etiology, consistent with granulomatous tissue changes (25). This combination effectively addresses the clinical overlap between GLM and DCIS, enhancing diagnostic precision in challenging cases. The nomogram’s reliance on widely available ultrasound and basic clinical data further positions it as a cost-effective alternative to advanced imaging modalities like MRI or deep learning radiomics, which are often inaccessible in resource-limited settings (9, 26).

The inclusion of tumor markers (CEA, CA125, CA153) in our analysis was based on their potential differential expression in inflammatory versus neoplastic breast conditions. However, our results demonstrated no significant differences in these markers between GLM and DCIS patients (**Table 1**), which has important clinical implications. This finding suggests that conventional tumor markers are inadequate for differentiating these conditions, supporting the need for our ultrasound and clinical feature-based nomogram. The lack of discriminatory value of these markers may be attributed to the fact that GLM, despite being benign, can elicit inflammatory responses that may affect marker levels, while early-stage DCIS may not significantly elevate these markers. This observation reinforces that morphological and clinical features, rather than serological markers, are more reliable for this specific differential diagnosis.

Our ROC curve analysis revealed that WBC count has moderate discriminatory value for differentiating GLM from DCIS, with an optimal cut-off of 7.735 × 10^9^/L and an AUC of 0.69. The elevation of WBC in GLM patients reflects the chronic inflammatory nature of this condition, which typically triggers a systemic immune response. While this biomarker provides useful supplementary information with reasonable sensitivity (68%) and positive predictive value (72%), our multivariable analysis demonstrated that imaging features offer superior discriminatory power, explaining why WBC was not retained in the final nomogram model. Nevertheless, the established cut-off value may serve as a valuable adjunct in clinical decision-making, particularly when combined with clinical and imaging findings, especially considering that WBC levels above 7.735 × 10^9^/L increase the likelihood of GLM diagnosis by 72%.

Despite these strengths, several limitations merit attention. The retrospective, single-center design at Quanzhou First Hospital may introduce selection bias, and the sample size of 290 patients, while adequately powered (128 GLM, 162 DCIS), is smaller than some multicenter cohorts, potentially limiting generalizability (27). Unassessed confounding factors, such as lifestyle variables (e.g., smoking, diet) (28), hormonal influences (e.g., estrogen levels) (28), and lactation-related factors (e.g., breastfeeding patterns, milk stasis) (29), could affect lesion characteristics and model performance, though their impact remains speculative due to data unavailability. The predominance of distinct margins in GLM (50.59% vs. 24.58% in DCIS) in our cohort, which differs from some previous reports, may be attributed to the higher proportion of cases with abscess formation in our series, as abscesses tend to develop well-defined boundaries that are interpreted as distinct margins. Therefore, the cohort’s representation of atypical GLM cases (e.g., those without abscess formation) is unclear, which may restrict applicability to diverse clinical presentations. Although internal validation demonstrated good performance with minimal overfitting, the lack of external validation remains a major limitation. External validation using independent multi-center datasets is essential to confirm the model’s generalizability and clinical applicability before widespread implementation.

Compared to existing models, this nomogram outperforms ultrasound and MRI-based discrimination models for differentiating non-lactational mastitis from breast cancer, which reported an AUC of 0.920 (26), by leveraging accessible ultrasound data without requiring advanced resources. It also rivals ultrasound-based deep learning models, which achieved a sensitivity of 0.92 but an AUC of approximately 0.90 (9), while offering a simpler, clinician-friendly approach. This aligns with the practical diagnostic needs highlighted in GLM literature (4). The decision curve analysis further supports its clinical utility, with net benefits exceeding “treat all” or “treat none” strategies.

Future research should focus on prospective validation of the nomogram in larger GLM cohorts and integration of advanced imaging techniques, such as contrast-enhanced ultrasound and elastography, which have shown promise in characterizing inflammatory breast conditions (30, 31).Furthermore, collaborative multi-institutional studies are warranted to establish the nomogram’s robustness across different healthcare settings and patient demographics.

## Conclusion

This study presents the first nomogram tailored for differentiating granulomatous lobular mastitis (GLM) from ductal carcinoma *in situ* (DCIS), achieving high diagnostic accuracy with an area under the curve (AUC) of 0.95 in the training set and 0.93 in the validation set. By integrating age, lesion size, margin characteristics, microcalcifications, posterior acoustic enhancement, and peri-lesional flow, the model offers a reliable, cost-effective tool that outperforms conventional BI-RADS ultrasound assessments, potentially reducing unnecessary biopsies significantly. Its reliance on widely available ultrasound and clinical parameters makes it particularly valuable in resource-limited settings, such as community hospitals. However, external validation in independent cohorts is required before clinical implementation.

## Data Availability

The raw data supporting the conclusions of this article will be made available by the authors, without undue reservation.
